# Investigation on the Bending Mechanism of Single-Crystal Copper Under High Bending Rates via Molecular Dynamics

**DOI:** 10.3390/mi16030314

**Published:** 2025-03-07

**Authors:** Peng Wu, Pengyue Zhao, Zhengkun Li, Jianwei Wu, Jiubin Tan

**Affiliations:** 1 Center of Ultra-Precision Optoelectronic Instrumentation Engineering, Harbin Institute of Technology, Harbin 150001, China; 23s136325@stu.hit.edu.cn (P.W.); wujianwei@hit.edu.cn (J.W.); jbtan@hit.edu.cn (J.T.); 2 Key Laboratory of Ultra-Precision Intelligent Instrumentation, Ministry of Industry Information Technology, Harbin 150080, China; 3 Key Laboratory of Electrical Quantum Standards for State Market Regulation, National Institute of Metrology, Beijing 100029, China

**Keywords:** flexible hinge, bending, microstructural evolution, internal stress, molecular dynamics

## Abstract

Leaf spring-type flexible hinges serve as critical transmission components in kilogram quantization energy balance systems. Investigating their bending behavior is crucial for enhancing measurement accuracy and ensuring structural reliability. This work employs molecular dynamics simulations to analyze the mechanical properties and deformation characteristics of such hinges under varying bending rates. The findings reveal a significant correlation between the bending rate and the hinges’ plastic deformation and microstructural evolution, indicating the presence of a critical bending rate. When the bending rate is below the critical threshold, the hinges exhibit excellent structural stability, characterized by low dislocation density, reduced von Mises stress, and limited temperature rise, making them suitable for long-term use. Conversely, when the bending rate exceeds the critical threshold, the hinges undergo significant plastic deformation, including notable increases in stress and temperature concentration, as well as microstructural alterations. Specifically, the initially stable crystal structure is disrupted, leading to the formation of numerous defect structures. These changes result in localized instability and elevate the risk of fatigue damage. This work comprehensively elucidates the mechanical responses and failure mechanisms of flexible hinges, providing valuable data and guidance for their optimized design and application.

## 1. Introduction

**Figure 1:**
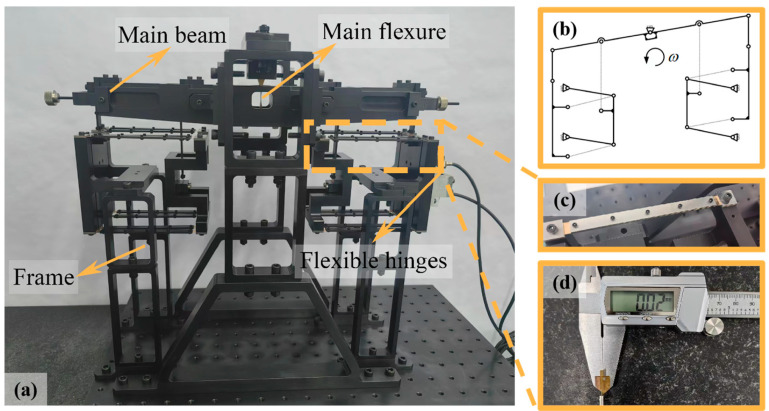
Structural diagram of the force measurement, guiding mechanism, and leaf spring-type flexible hinges in the energy balance system: (**a**) overall schematic diagram of the energy balance system, (**b**) schematic diagram of the motion principle, (**c**) magnified view of one of the 13-level flexible hinges, and (**d**) measurement of the 20 μm thickness of the leaf spring using a vernier caliper.

The energy balance system, as one of the devices for realizing kilogram quantization, operates based on the principle of conservation and conversion of electromagnetic energy, gravitational potential energy, and mechanical energy to replicate the kilogram [1,2]. During testing, the energy balance achieves equilibrium between the electromagnetic force generated by the coil and the gravitational force of the test weight through the reciprocal motion of the force measurement and guiding mechanism [3], as illustrated in Figure 1a. The reciprocating motion on both sides of the mechanism is achieved through the coupled motion of the 13-level hinges, as shown in the schematic diagram in Figure 1b. Considering that the rotational gaps in traditional hinges can reduce motion accuracy, all 13 hinges are designed as flexible hinges, as depicted in Figure 1c.The specific structure of the flexible hinges is a leaf spring, with thicknesses ranging from 20 μm to 50 μm processed using electrical discharge wire cutting technology, as shown in Figure 1d. These leaf spring flexible hinges enable the force measurement and guiding mechanism to achieve a stiffness below 5 N/m and a motion linearity better than 5 μm through complex coupled motion [4,5]. This work focuses on two critical aspects of leaf spring-type flexible hinges:

(I) The material’s plastic deformation and microstructural evolution during the bending process of the flexible hinges;
(II) The effect of the bending rate on the deformation behavior of the flexible hinges.

Research on flexible hinges primarily includes experimental and simulation methods. Experimental methods focus on validating the mechanical properties and structural optimization of flexible hinges through physical testing, such as cross-blade flexible hinges and composite spring materials. These methods are commonly employed to explore the mechanical properties and structural optimization of flexible hinges. In terms of shape optimization, Gómez et al. [6] examined the shape optimization of cross-blade flexible hinges, achieving significant reduction in maximum stress through thickness contour optimization, thereby extending fatigue life. Qiu et al. [7] proposed a design method for contact-assisted blade hinges that enables a transition from discrete to continuous stiffness, validating the design’s feasibility through experiments and exploring parasitic motion and coupling effects. For experimental validation in thin-walled structures, Oberst et al. [8] demonstrated the performance of thin-walled band springs under static and vibrational loads through ground-based experiments, analyzing the inverse relationship between damping and deployment length. Thaker et al. [9] reviewed band springs in deployable space structures, discussing key issues such as folding and deployment analysis, strain energy storage, and stability, offering guidance for future research. In terms of damping and dynamic control, Chen et al. [10] introduced a blade hinge model with a damping layer and experimentally verified the effect of different damping configurations on vibration suppression, showing that constrained layer damping can significantly enhance hinge damping characteristics. Meijaard et al. [11] studied the guiding system of parallel-blade springs, highlighting that over-constraints and manufacturing errors cause notable changes in static and dynamic characteristics, with slight misalignment affecting system stiffness and natural frequency. In composite blade development, Guduru et al. [12] investigated the mechanical properties of composite monolithic springs, finding that glass and carbon fiber composites substantially reduced the weight while maintaining elasticity and load capacity. Saelem et al. [13] confirmed the nonlinear deformation of blade springs through experimental apparatus, revealing a lag effect in static offset due to the loading direction, thus providing insights for vehicle suspension system design. For complex flexible structure design, Ghuku et al. [14] developed a testing apparatus for blade springs and, through image processing, measured the deformation profile of primary blades, identifying current model limitations and suggesting improvements. Kang et al. [15] presented a design and analytical model for wheel-type flexible hinges, verifying the accuracy of its stiffness model in finite-element simulations and finding that manufacturing errors notably affected the predictive accuracy. However, experimental methods are often time-consuming and costly, and they are limited in their ability to thoroughly analyze internal stress distribution, microstructural changes, and dynamic deformation phenomena in hinges, particularly for precise characterization at small scales or in complex structures.

Simulation studies on small-scale flexible hinges typically employ molecular dynamics (MD) methods to elucidate material behavior under varying conditions, thereby providing theoretical support for flexible structural design. Regarding the fundamental mechanical properties of materials, Borysiuk et al. [16] investigated the bending stiffness of nanoribbons and found that their stiffness increases nonlinearly with the load until reaching a critical point. Cao et al. [17], through systematic MD and finite-element method (FEM) simulations, studied the bending–buckling behavior of thin-walled structures, analyzing the relationships between critical buckling curvature, critical strain, and structural parameters. They pointed out that traditional continuum-shell models fail to quantitatively reproduce the bending–buckling behavior of such micro-scale structures. Ru et al. [18] proposed that the effective bending stiffness of thin layers should be considered an independent material parameter. Zepeda-Ruiz et al. [19] demonstrated that, when dislocations no longer bear the load, deformation twinning becomes the primary response mechanism in crystalline metals. Chen et al. [20] investigated the structure–mechanical relationship in elastic materials, revealing that molecular interactions significantly influence stress–strain behavior, providing a new perspective for understanding elasticity. In terms of temperature and loading rate effects, Reddy et al. [21] explored the impact of temperature on the fracture behavior of nanocrystalline metals, discovering that, with increasing temperature, the fracture mode of the material shifts from brittle to ductile. Deb Nath et al. [22], using MD simulations, examined the mechanical properties of metallic nanowires under varying temperatures and sizes, showing that Young’s modulus and yield strength are influenced by these factors. Wu et al. [23] studied the bending behavior of metallic rods under different loading rates, showing that surface effects cause a deviation in mechanical properties from classical predictions. For multilayer structures and interlayer interactions, Shen et al. [24] studied the effect of interlayer shear on the bending stiffness of multilayer crystalline structures, finding that the shear modulus predominantly governs the bending behavior. Wei et al. [25] suggested that the bending stiffness of ultra-thin metallic layers closely resembles that of biological membranes, potentially supporting applications where flexibility and strength are needed. Fan et al. [26] examined the deformation modes of crystalline microstructures under various loading conditions by altering crystallographic orientation, revealing a significant impact of the lattice orientation on the bending behavior. In the context of complex deformation behaviors in flexible structures, Tian et al. [27] investigated the deformation mechanism of crystalline nanowires under bending and torsion, observing that the formation of penta-twinned structures is closely related to stress direction. Horx et al. [28] analyzed the dynamic behavior of hinge-like peptide structures, revealing that the opening angle and twist mode are the primary dynamic characteristics. Lu et al. [29], through first-principle MD, studied the bending ductility of metallic glass composites, finding that specific compositional adjustments can significantly enhance magnetic properties and ductility.

This work focuses on plate spring flexible hinges primarily made of copper, utilizing MD simulations to analyze their atomic-scale deformation behavior and mechanical properties under various bending rate conditions. Although the simulation scale does not match the larger practical scale of flexible hinges, this approach is essential as traditional FEM simulations are less effective at such small scales. The insights gained are crucial for establishing a foundational understanding of material behaviors under stress and guiding the design of flexible structures in engineering applications.

## 2. Materials and Methods

**Figure 2:**
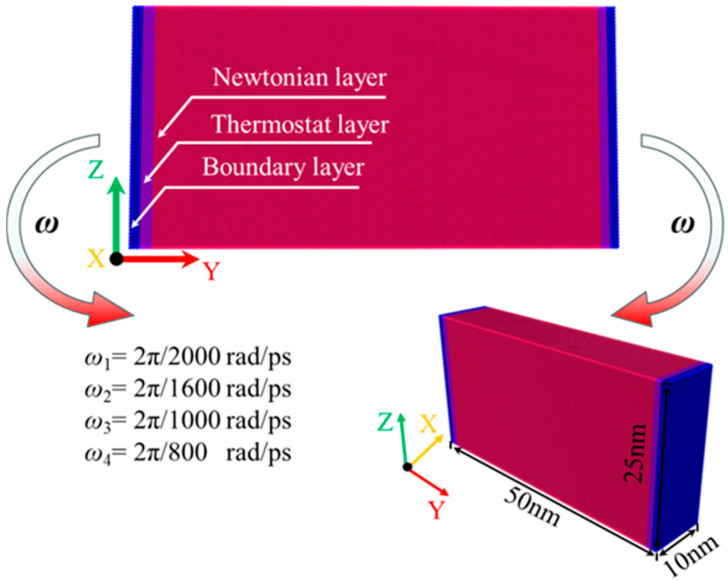
Structural diagram of the force measurement, guiding mechanism, and leaf spring-type flexible hinges in the energy balance system.

The molecular dynamics (MD) bending simulation model of the flexure-type flexible hinge, as shown in Figure 2, used single-crystal copper (Cu) with a lattice constant of 3.615 Å. The MD model dimensions were 100 Å × 500 Å × 250 Å, containing approximately 1,066,464 atoms. Although this scale was substantially smaller than that of actual flexible hinges, the micro-mechanisms studied still provided a basis for understanding the behavior of similar materials in macro-applications. The crystal orientations along the X-, Y-, and Z-axis of the MD model were [100], [010], and [001], respectively. The bending simulation model was divided into three regions: the Newton layer, the thermostat layer, and the fixed layer. In the Newton layer, atoms underwent primary deformation, following Newton’s laws, using the velocity Verlet method [30]. The thermostat layer maintained a constant temperature of 293 K, regulated by the Berendsen thermostat [31]. Atoms in the fixed layer retained the initial parameters to support the system. To minimize size effects, periodic boundary conditions (PBCs) were applied in the X-, Y-, and Z-axis.

**Figure 3:**
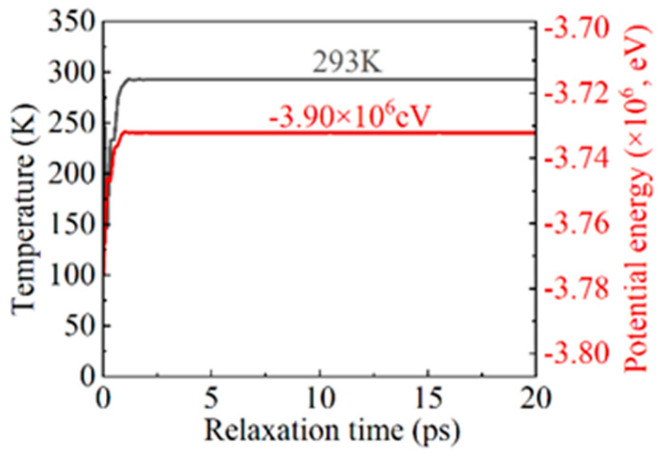
Temperature and total potential energy curves during the relaxation process.

The MD simulation of bending primarily included the relaxation phase and the bending phase. In the relaxation phase, the system underwent energy minimization using the conjugate gradient method [32], followed by relaxation in an isothermal–isobaric ensemble (NPT) at a temperature of 293 K and a pressure of 1 atm for a duration of 20 ps.The relaxation results, shown in Figure 3, indicated a gradual decrease in both the system temperature and the total potential energy until stabilization, with the temperature stabilizing at 293 K and the total potential energy reaching −3.90 × 106 eV. This suggests that, during relaxation, the system gradually attained a stable state as the microstructure adapted, reducing local stress concentrations to achieve equilibrium. In the bending phase, the model underwent a bending simulation in a constant-energy ensemble (NVE) with an applied bending torque. The two ends rotated at opposing angular velocities, with angular velocities of *ω*_1_ = ±2π/2000 rad/ps, *ω*_2_ = ±2π/1600 rad/ps, *ω*_3_ = ±2π/1200 rad/ps, and *ω*_4_ = ±2π/800 rad/ps, where *ω*_1_ through *ω*_4_ progressively increased. The bending axis was centered on the model and aligned parallel to the X-axis. The bending phase durations were set to 200 ps, 160 ps, 120 ps, and 100 ps for each respective bending rate, ensuring a total bending angle of 2π/5 rad (referred to as *T* for the complete bending process in this work). This angle range satisfied the requirements for analyzing the full operational and failure behavior of plate spring-type flexible hinges. A timestep of 1 fs was used, and strain and mean square displacement (MSD) were continuously monitored and recorded throughout the bending process. The bending simulation MD model was executed using the Large-scale Atomic/Molecular Massively Parallel Simulator (LAMMPS) [33]. The MD simulation parameters are provided in Table 1. In addition, the simulated file data were visualized and analyzed by OVITO.

The MD simulation utilized the embedded atom method (EAM) potential function [34] to model the interactions between copper atoms. The EAM potential calculated the total energy by combining the pair potential between the atoms and the embedding energy of an atom within the surrounding electron density. This approach is particularly suited for simulating metallic materials, as it effectively captures complex interatomic interactions.

(1) $$\left\{\begin{array}{c} E_{\mathrm{total}}=\sum_{i}F(\rho_{i})+\sum_{i\neq j}\phi (r_{ij})/2\\ \rho_{i}=\sum_{i\neq j}f(r_{ij}) \end{array}\right.,$$

where *E*_total_ represents the system’s total energy, *F*(*ρ_i_*) denotes the embedding energy of atom *i* in the local electron density *ρ_i_*, *ϕ*(*r_ij_*) is the pair potential between atoms *i* and *j*, which depends on the distance *r_ij_* between them, and *f*(*r_ij_*) is a function that contributes to the electron density at distance *r_ij_*. In this work, dislocations were identified and quantified using the dislocation extraction algorithm (DXA) available in the OVITO software (Basic-3.9.2) [35]. The Lagrangian finite-strain tensor [36] was employed to describe the plastic strain of the material, which is suitable for capturing nonlinear deformations, expressed by the following equation:

(2) $$E=\frac{1}{2}(F^{T}F-I),$$

where **E** represents the Green–Lagrange strain tensor, **F** denotes the deformation gradient tensor, and **I** is the identity matrix. Hydrostatic stress [37] described the state where a point within a material experienced equal tensile stress in all directions, defined by the following equation:

(3) $$\sigma_{hyd}=(\sigma_{xx}+\sigma_{yy}+\sigma_{zz})/3,$$

where *σ_hyd_* represents the hydrostatic stress, and *σ_xx_*, *σ_yy_*, and *σ_zz_* denote the normal stress components. The von Mises stress [38] is a widely utilized metric in metal plastic deformation and yield criteria, representing the equivalent stress under complex stress conditions. It is given by the following formula:

(4) $$\sigma_{v}=\sqrt{[{\left(\sigma_{xx}-\sigma_{yy}\right)}^{2}+{\left(\sigma_{yy}-\sigma_{zz}\right)}^{2}+{\left(\sigma_{zz}-\sigma_{xx}\right)}^{2}+6(\tau_{xy}^{2}+\tau_{yz}^{2}+\tau_{zx}^{2})]/2},$$

where *σ_v_* is the von Mises stress, and *τ_xy_*, *τ_yz_*, and *τ_zx_* represent the shear stress components. The temperature distribution in each region of the system was estimated based on the kinetic energy of the system atoms [39], with calculations depending on the total kinetic energy and the degrees of freedom, as expressed by the following:

(5) $$\left\{\begin{array}{c} Te=\frac{2KE}{(3N-f)k_{B}}\\ KE=\sum_{i=1}^{N}KE_{i}=\sum_{i=1}^{N}m_{i}(v_{ix}^{2}+v_{iy}^{2}+v_{iz}^{2})/2 \end{array}\right.,$$

where *Te* is the temperature, *KE* is the system’s total kinetic energy, *f* is the number of constrained degrees of freedom, *k_B_* is the Boltzmann constant, and *m_i_* and *v_ix_*, *v_i__y_* and *v_i__z_* denote the mass and velocity components of the *i*-th atom in the X-, Y-, and Z-axis, respectively.

## 3. Results

### 3.1. Plastic Deformation Process

**Figure 4:**
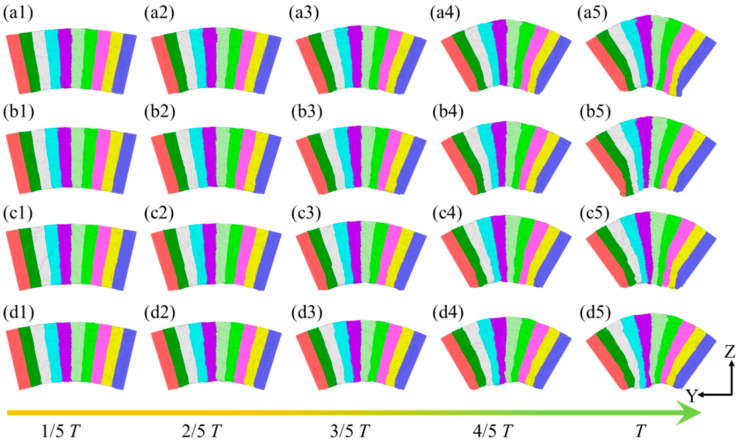
Plastic deformation process at different positions in the leaf spring-type flexible hinge under various bending rates, with (**a1**–**d5**) representing *ω*_1_ = 2π/2000 rad/ps, *ω*_2_ = 2π/1600 rad/ps, *ω*_3_ = 2π/1200 rad/ps, and *ω*_4_ = 2π/800 rad/ps, respectively.

Figure 4 illustrates the plastic deformation of plate spring flexible hinges under different bending rates. For the ease of observation and comparison, the model is divided into 10 equal sections along the Z-axis. The results indicate that, despite variations in the bending rate, the overall deformation trend of the system remains consistent across four different rates. As the bending angle increases, the atoms at the top of the sample progressively extend, while those at the bottom undergo compression and extrusion, resulting in irregular curvatures and atomic accumulation. Horstemeyer et al. [40] studied the plastic response of single-crystal copper under stress via bending experiments, revealing that stress can induce deformation wave propagation in materials, a phenomenon comparable to the plastic deformation process of the flexible hinge at different bending rates in this work. At the bending rate *ω*_1_, as shown in Figure 4a, initial deformation in the system is observed during the 0~1/5 *T* phase. In the subsequent 2/5 *T*~3/5 *T* bending stage, atomic extension at the top of the sample continues, while atoms at the bottom further compress, forming a relatively smooth bending curvature. As the bending angle reaches 4/5 *T*~*T*, the atomic extension at the top and compression at the bottom become more pronounced, with the bottom curvature tending toward irregularity. A similar deformation behavior is also observed under higher bending rates, such as *ω*_3_ and *ω*_4_, as depicted in Figure 4(c5,d5).

**Figure 5:**
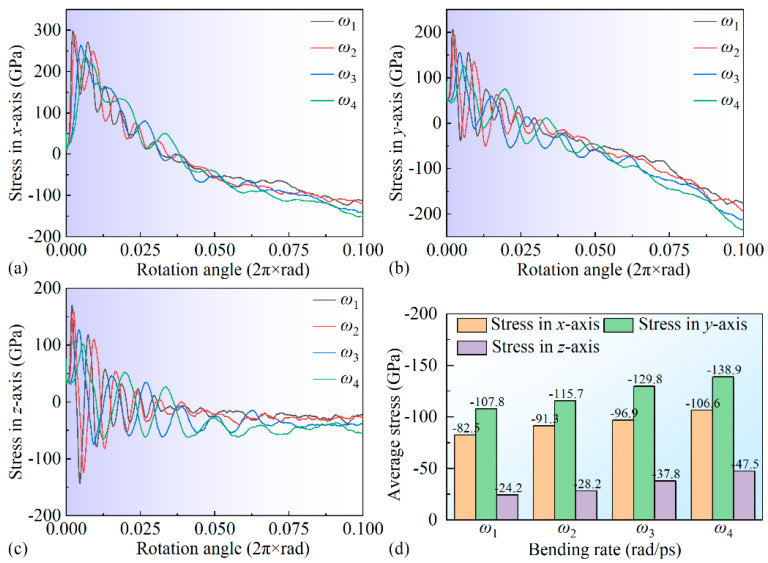
Stress variation curves in different directions for the leaf spring-type flexible hinge under various bending rates: (**a**) X-axis stress, (**b**) Y-axis stress, (**c**) Z-axis stress, and (**d**) stress statistics.

Figure 5 illustrates the stress variation curves of the leaf spring-type flexible hinge under different bending rates, where positive values indicate tensile stress, and negative values indicate compressive stress. During bending, the tensile stress along the X-axis gradually increases in the 0~1/5 *T* phase, reaching a peak of approximately 300 GPa, before gradually decreasing. Around 1/2 *T*, the tensile stress transitions to compressive stress, which continues to increase during bending and ultimately reaches around −120 GPa. This finding indicates a significant transition between tensile and compressive stress in the flexible leaf spring hinge during bending, likely a critical factor contributing to failure in its operational use. The Y-axis stress follows a similar pattern, exhibiting a transition from tensile to compressive stress around 1/3 *T*, after which the compressive stress gradually increases, ultimately reaching approximately −200 GPa. Notably, this direction also shows a pronounced transition between tensile and compressive stress. Stress–strain curves in the X-, Y-, and Z-axis reveal distinct stress oscillations associated with dislocation slip due to crystalline structural defects within the material. These high-stress regions may be a primary cause of fatigue failure in the flexible hinge, especially in areas of frequent stress oscillations, where stress concentration leads to cumulative structural damage. Averaging the stress–strain curves across all three axes yields the results shown in Figure 5d, indicating that the average stress in all three directions during bending primarily exhibits compressive stress, which increases significantly with rising bending rates.

**Figure 6:**
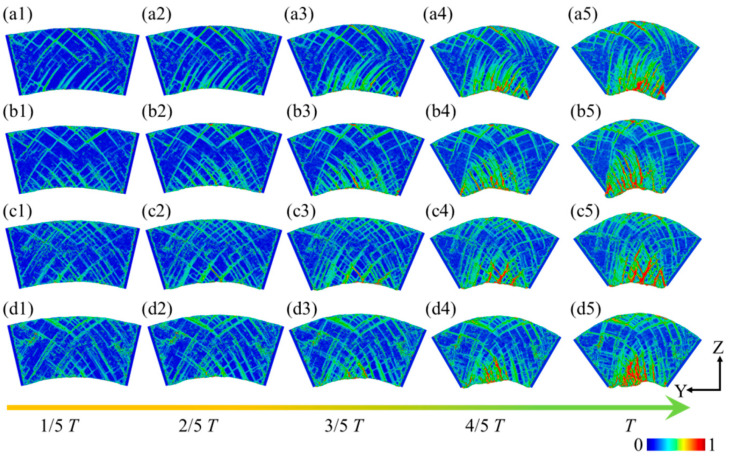
Strain distribution in the leaf spring-type flexible hinge under different bending rates, with (**a1**–**d5**) corresponding to *ω*_1_ = 2π/2000 rad/ps, *ω*_2_ = 2π/1600 rad/ps, *ω*_3_ = 2π/1200 rad/ps, and *ω*_4_ = 2π/800 rad/ps.

Figure 6 illustrates the strain distribution of a leaf spring-type flexible hinge under different bending rates. Significant strain is observed during the bending process of the flexible hinge. As the bending degree increases, numerous intersecting high-strain regions emerge within the system. These areas are primarily characterized by stacking fault structures along the (111) slip plane in the [110] crystal orientation, as shown in Figure 6(a1). With further bending, the internal strain regions expand with the increased bending degree, and more pronounced strain appears in areas of greater curvature beneath the sample, as shown in Figure 6(a3). Eventually, prominent red strain concentration areas emerge at the bottom and top of the system, as depicted in Figure 6(a5). Throughout the bending process, the strain at both ends of the system remains low. As the bending rate increases, internal strain intensifies further, with the strain concentration areas at the system’s bottom and top expanding, as seen in Figure 6(b5–d5). This indicates that higher bending rates lead to greater internal strain within the system, resulting in a more pronounced stress concentration, which could potentially cause fatigue damage to the leaf spring-type flexible hinge during operation.

### 3.2. Microstructure Evolution

**Figure 7:**
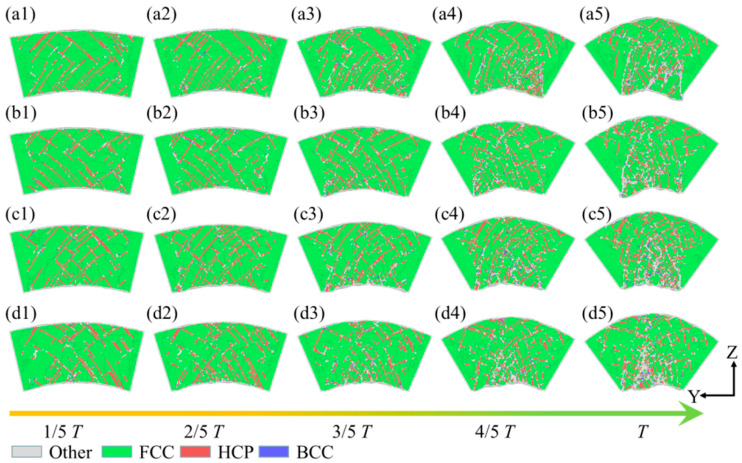
Defect evolution within the leaf spring-type flexible hinge under various bending rates, with (**a1**–**d5**) corresponding to *ω*_1_ = 2π/2000 rad/ps, *ω*_2_ = 2π/1600 rad/ps, *ω*_3_ = 2π/1200 rad/ps, and *ω*_4_ = 2π/800 rad/ps.

During the bending simulation, the effect of different bending curvatures on the structural evolution within the curved segments of the plate spring flexible hinge was analyzed across various bending rates. Figure 7 presents the evolution of the internal structure of the plate spring flexible hinge under different bending rates and curvatures, analyzed using common neighbor analysis (CNA). When the bending curvature reached 1/5 *T*, the stable FCC crystal structure within the hinge experienced orientational rearrangement under stress, leading to the emergence of defect structures with HCP crystal patterns in the middle and on both sides of the hinge. These defects, primarily represented by intrinsic stacking faults (ISFs) and extrinsic stacking faults (ESFs), slid along the {111} crystal planes in the [110] direction, as shown in Figure 7(a1). This structural transformation indicated a local reduction in the material’s resistance to tensile stress, potentially leading to instability and fatigue damage. With an increasing curvature, the internal curved structures in the plate spring flexible hinge grew more prevalent, and stacking faults continued to expand inward. When the curvature reached 4/5 *T*, amorphization appeared at the bottom of the specimen, forming an amorphous structure as FCC atoms decreased further, as shown in Figure 7(a4). At full curvature (T), amorphous structures became more prominent, and the FCC crystal structure further diminished, as seen in Figure 7(a5). At higher bending rates, although fewer internal structures were initially observed at a bending curvature of 1/5 *T*, the internal structure progressively increased with curvature, with significant amorphous formation at full curvature, as shown in Figure 7b. With higher bending rates, the internal structure within the plate spring flexible hinge markedly increased as the curvature grew, with stacking faults spreading further into the material and FCC atoms continuously decreasing, and this may have also caused fatigue damage to the leaf spring flexible hinge during operation. As the bending rate increased, the evolution of the internal structure exhibited notable changes, with more pronounced differences as the curvature intensified, as illustrated in Figure 7c,d.

**Figure 8:**
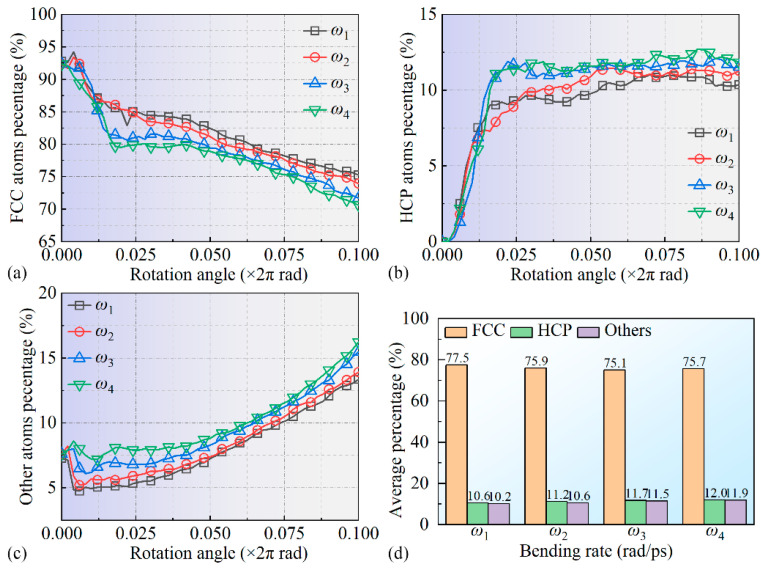
Changes in the atomic crystal structure of the leaf spring-type flexible hinge during bending, showing ratios of atoms in FCC, HCP, and amorphous structures under different bending rates (**a**–**d**).

Figure 8 shows the variation curves of atomic ratios with amorphous, FCC, and HCP crystal structures under different bending rates. Note that the BCC crystal structure atoms present in the system were omitted during statistical analysis. It was observed that, as bending progressed, the trend in percentage changes in atoms with FCC, HCP, and amorphous structures remained generally similar under different bending rates, though the degree of change varied. During the 0–1/5 *T* phase, the proportion of FCC structure atoms in the system decreased significantly, indicating that the stable FCC crystal structure was disrupted due to the surface’s bending process. Zheng et al. [41] also noted structural instability and amorphization in crystals under stress, which may explain the observed FCC-to-amorphous transformation trend under the high bending rates in this work. Notably, the changes in *ω*_3_ and *ω*_4_ were more pronounced than in *ω*_1_ and *ω*_2_, likely due to intensified atomic interactions at higher bending rates. Subsequently, in the 1/5 *T*~*T* phase, the proportion of FCC structure atoms in the system continued to gradually decrease, following an approximately linear trend, related to the continuous expansion of multilayer stacking faults, as shown in Figure 8a. At the end of the bending process, the residual FCC atomic ratios in the system were about 75%, 73%, 72%, and 71% for bending rates *ω*_1_, *ω*_2_, *ω*_3_, and *ω*_4_, respectively. During the 0–1/5 *T* phase, the percentage of HCP atoms within the system rapidly increased with bending, indicating a substantial transformation of FCC to HCP crystal structures, as shown in Figure 8a,b. In the 1/5 *T*~*T* phase, the percentage of HCP atoms stabilized, with maximum proportions of 10%, 11%, 11.5%, and 12% corresponding to bending rates *ω*_1_, *ω*_2_, *ω*_3_, and *ω*_4_, respectively, indicating that higher bending rates facilitated the formation and expansion of HCP defects, as shown in Figure 8b. Additionally, during the 0–1/5 *T* phase, the proportion of amorphous atoms remained low and changes minimally, while, in the 1/5 *T*~*T* phase, this proportion increased significantly, suggesting the amorphization of FCC atoms, as illustrated in Figure 8a,c. Higher bending rates further accelerated this amorphization process, as shown in Figure 8c. Xu et al. [42] found that dislocation activities under loading conditions could induce grain boundary sliding and structural transformation in nanocrystalline materials, consistent with the HCP and amorphous expansion trends observed under higher bending rates in this work. To quantify the crystal structure transformation process further, the crystal proportions during the 1/2 *T*~*T* phase were averaged, with the results presented in Figure 8d. These results indicate that intense FCC-to-HCP transformation occurred during the 0–1/5 *T* phase, while the FCC-to-amorphous transition dominated in the 1/5 *T*~*T* phase, with higher bending rates *ω*_3_ and *ω*_4_ further promoting both processes.

**Figure 9:**
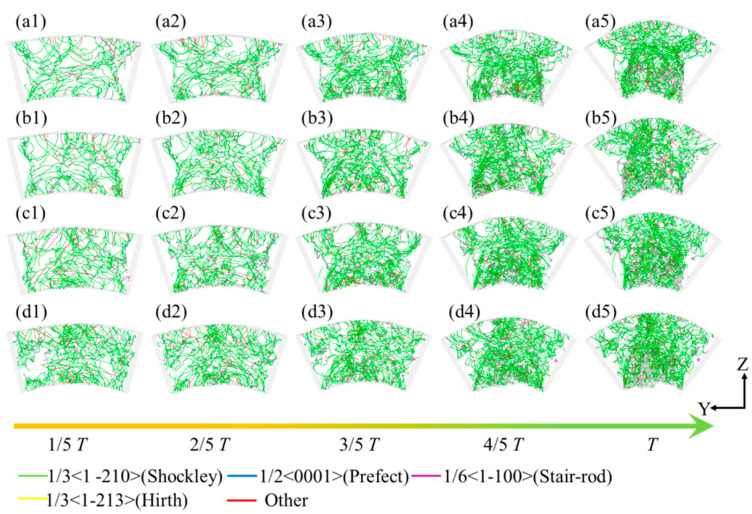
Evolution of dislocation lines within the leaf spring-type flexible hinge at different bending rates, with (**a1**–**d5**) corresponding to *ω*_1_ = 2π/2000 rad/ps, *ω*_2_ = 2π/1600 rad/ps, *ω*_3_ = 2π/1200 rad/ps, and *ω*_4_ = 2π/800 rad/ps.

Figure 9 illustrates the derived dislocation line configurations within the leaf spring-type flexible hinge during bending simulation, obtained using the dislocation extraction algorithm (DXA). In this figure, the green, purple, blue, yellow, and red lines represent Shockley partial dislocations (1/3<1-210>), stair-rod partial dislocations (1/6<1-100>), perfect dislocations (1/2<0001>), Hirth partial dislocations (1/3<1-213>), and other dislocation types, respectively. A substantial presence of Shockley partial dislocations, along with fewer stair-rod, perfect, and Hirth partial dislocations, was observed within the system during bending, as depicted in Figure 9(a1). This indicated significant plastic deformation and crystal structure transformation within the specimen. The emergence of Shockley dislocations may have resulted from slip at the interface of tensile and compressive stresses, with their increase possibly leading to localized instability in the material. Lock dislocations typically formed when two dislocations with different Burgers vectors intersect and react, creating a sessile dislocation which can act as an obstacle to further dislocation motion. This could lead to increased stress concentrations and potentially influence the material’s failure mechanisms. As the bending strain intensifies, dislocations accumulate predominantly at the system’s top and bottom regions, corresponding to areas of pronounced plastic deformation, as shown in Figure 9(a5). Furthermore, as the bending speed gradually increases, the number of dislocation lines within the system also rises, as observed in Figure 9b–d. Higher bending rates induce a more intense stress concentration, enhancing nonlinear deformation and dislocation multiplication within the material. This rate-dependent behavior may affect the fatigue life of the material and provide insights for designing flexible hinges suited to various loading conditions.

**Figure 10:**
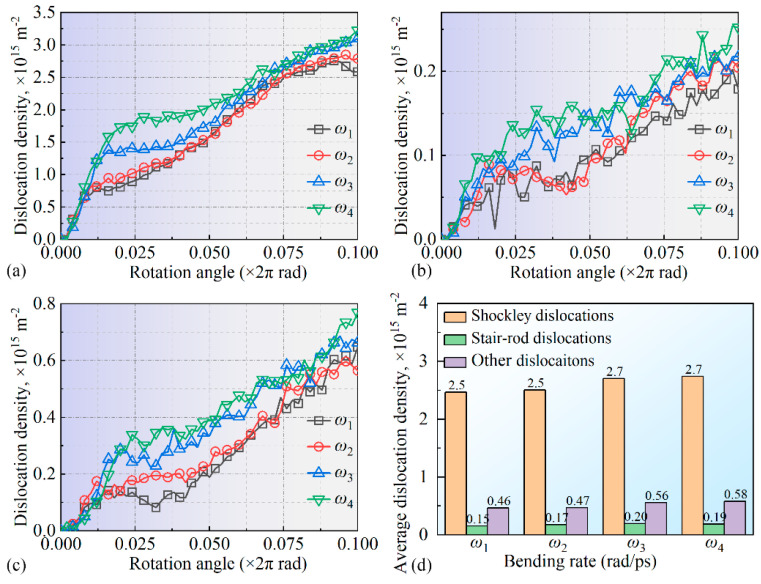
Variation in dislocation line density during the bending of the leaf spring-type flexible hinge: (**a**) Shockley partial dislocations, (**b**) stair-rod partial dislocations, (**c**) other dislocations, and (**d**) statistical results of average dislocation density.

To further quantify the internal dislocation line variations in the system, the densities of various dislocation types were calculated, with the results shown in Figure 10. Shockley partial dislocations (a) and stair-rod partial dislocations (b) were analyzed individually, while all other dislocation lines were grouped as “other dislocations” (c). During the bending process, a significant increase in Shockley partial dislocations was observed in the 0–1/5 *T* stage. Under bending rates *ω*_3_ and *ω*_4_, the dislocation line density rapidly rose to 1.8 × 10^15^ m^−2^ and 1.3 × 10^15^ m^−2^, respectively, much higher than the 0.8 × 10^15^ m^−2^ observed under bending rates *ω*_1_ and *ω*_2_, indicating substantial microstructural changes within the system at this stage. Research by Sharma et al. [43] showed that dislocation density increases rapidly with changing loading conditions, accompanied by marked microstructural changes, consistent with the rapid increase in Shockley dislocation density under higher bending rates observed in this work. In the 1/5 *T*~*T* stage, the Shockley partial dislocation growth trend showed a near-linear increase, matching the phenomenon seen in Figure 8. Additionally, higher bending rates were found to promote dislocation line density in Shockley partial dislocations. Zheng et al. [44] found that under high-frequency vibration, the internal dislocation structure of materials evolves significantly due to external loading changes, a phenomenon similar to the dislocation density increase observed at high bending rates in this work, indicating that the loading rates may significantly influence the microstructure within a system. For stair-rod partial dislocations, the density consistently increased throughout the bending process across all bending rates, with *ω*_3_ and *ω*_4_ yielding slightly higher densities than *ω*_1_ and *ω*_2_, as shown in Figure 10b. Rajgarhia et al. [45] noted that, in plastic deformation, dislocation formation and accumulation are closely related to the loading rate, with higher rates accelerating dislocation density growth. This finding supports the increased stair-rod dislocation density observed under higher bending rates in this work. A similar trend is observed in Figure 10c for other dislocations, where higher bending rates promote density increases across dislocation types. To further quantify the dislocation line density evolution, the average densities of various dislocation types during the 1/2 *T*~*T* bending phase were calculated, as shown in Figure 10d. The results indicate that the system produced a substantial number of Shockley and stair-rod partial dislocations during the 0–1/5 *T* stage of bending, with a gradual increase in the dislocation line density throughout the process. Higher bending speeds further promoted dislocation line density accumulation within the system.

### 3.3. Internal Stress

**Figure 11:**
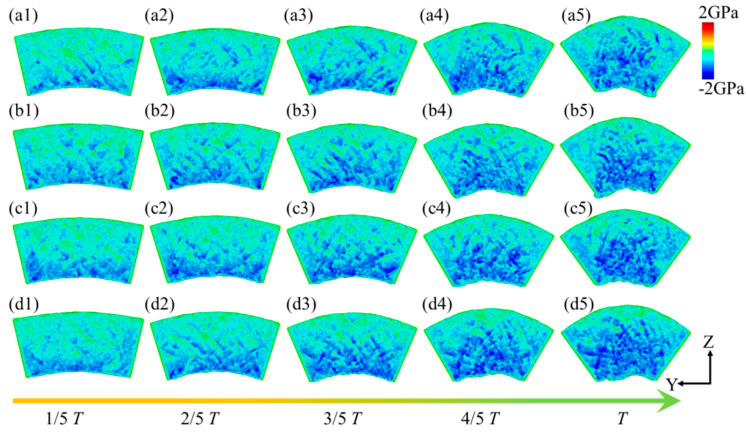
Hydrostatic stress distribution within the leaf spring-type flexible hinge under different bending rates, with (**a1**–**d5**) corresponding to *ω*_1_ = 2π/2000 rad/ps, *ω*_2_ = 2π/1600 rad/ps, *ω*_3_ = 2π/1200 rad/ps, and *ω*_4_ = 2π/800 rad/ps.

**Figure 12:**
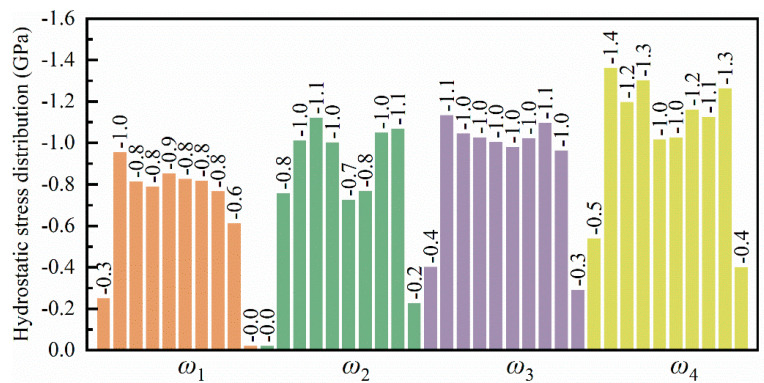
Statistical distribution of average hydrostatic stress in the leaf spring-type flexible hinge after bending under different bending rates.

Figure 11 illustrates the hydrostatic stress distribution within a leaf spring-type flexible hinge under various bending rates, with red indicating tensile stress and blue indicating compressive stress. The system exhibits generally low tensile stress throughout the bending process; however, a significant compressive stress region emerges at the system’s bottom as bending increases. Guo et al. [46] observed that the stress concentration within a microstructure is closely associated with the material’s stress distribution, which may explain the concentrated compressive stress observed at the bottom during bending in this work. Under such conditions of high compressive stress, the microstructure of the material may progressively destabilize, potentially leading to microcracks or fractures, particularly in copper-based materials where stress release under high pressure can be accompanied by plastic deformation or increased crystal defects. As bending increases further, the compressive stress region gradually expands toward the central and end areas of the system, resulting in tensile stress at the top of the system, with a tensile stress value of approximately 1.0 GPa, while the central and bottom areas mainly experience compressive stress ranging from −2.2 GPa to −2.9 GPa, as shown in Figure 11a. With an increase in bending rate, the accumulation and distribution range of compressive stress within the system tend to intensify, consistent with the stress–strain pattern observed in Figure 5, attributed to more intense strain between the material atoms at higher bending rates. Zhao et al. [47] demonstrated that microstructural changes under high-stress conditions significantly affect local stress distribution, aligning with the trend of expanded compressive stress distribution observed in this work at higher bending rates. For different bending rates, the average hydrostatic stress along the Y-axis direction within the system’s 10 internal divisions at the bending limit position is shown in Figure 12. All regions within the system exhibit compressive stress, with lower compressive stress at the ends and higher compressive stress in the central region, indicating a relatively uniform stress distribution. Moreover, as the bending rate increases, a noticeable rise in hydrostatic stress across all regions within the system is observed, increasing from −0.8 GPa to −1.3 GPa, with a growth of approximately 27.8%.

**Figure 13:**
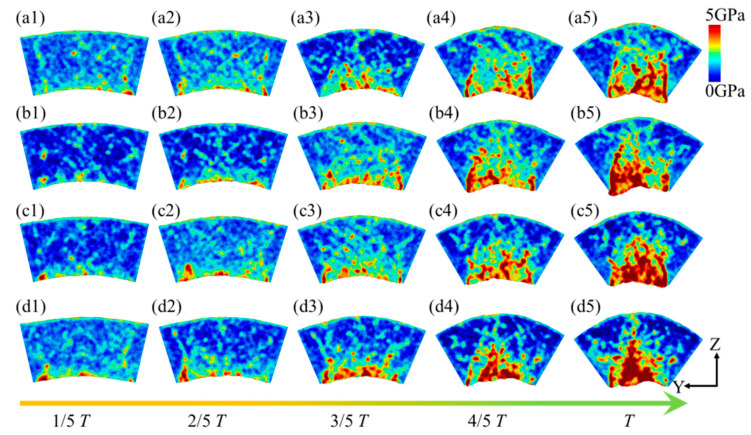
Distribution of von Mises stress in the leaf spring-type flexible hinge under various bending rates, with (**a1**–**d5**) representing *ω*_1_ = 2π/2000 rad/ps, *ω*_2_ = 2π/1600 rad/ps, *ω*_3_ = 2π/1200 rad/ps, and *ω*_4_ = 2π/800 rad/ps.

**Figure 14:**
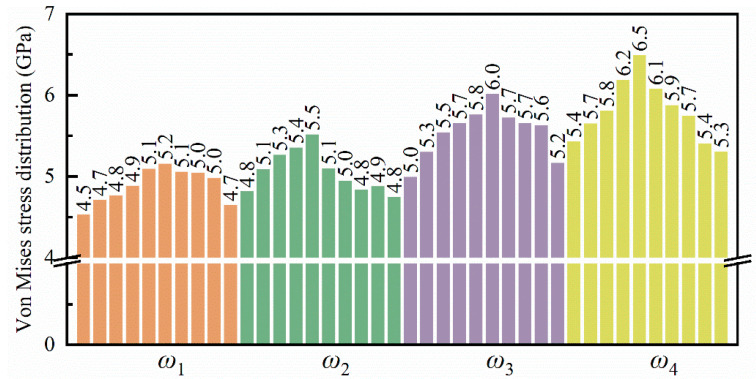
Statistical distribution of average von Mises stress in the leaf spring-type flexible hinge after bending under different bending rates.

Compared to hydrostatic stress, von Mises stress comprehensively considers six stress components, which can more comprehensively characterize the stress state inside the workpiece. Figure 13 illustrates the von Mises stress distribution within the flexure hinge of the leaf spring type under varying bending rates. During the bending process, high-stress regions are primarily concentrated at the system’s base. As bending progresses, these high-stress areas gradually extend toward the central and upper sections of the system. The range of high-stress regions expands accordingly, with values ranging from approximately 4.7 GPa to 5.2 GPa. Moreover, higher bending rates lead to an increase in both the extent and rate of stress propagation, consistent with the observations in Figure 11. Figure 14 depicts the average von Mises stress distribution at the bending limit position within the system for different bending rates. It reveals that von Mises stress is lower at both ends of the system while being higher in the central region, forming a stress gradient. Additionally, with increased bending rates, there is a marked rise in the von Mises stress distribution within the system.

### 3.4. Temperature

**Figure 15:**
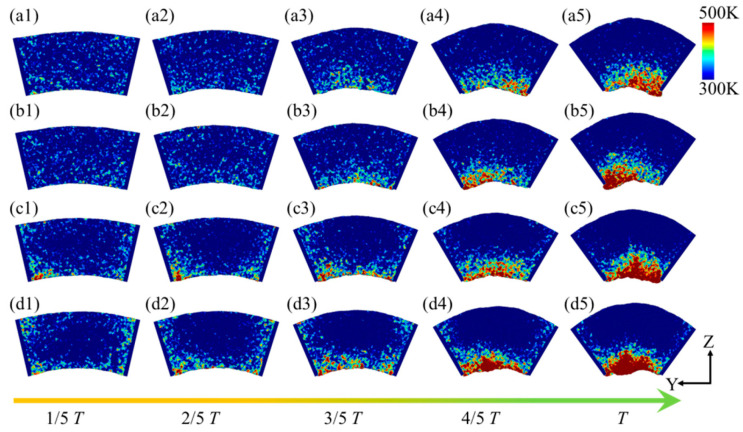
Temperature distribution within the leaf spring-type flexible hinge under various bending rates, with (**a1**–**d5**) corresponding to *ω*_1_ = 2π/2000 rad/ps, *ω*_2_ = 2π/1600 rad/ps, *ω*_3_ = 2π/1200 rad/ps, and *ω*_4_ = 2π/800 rad/ps.

**Figure 16:**
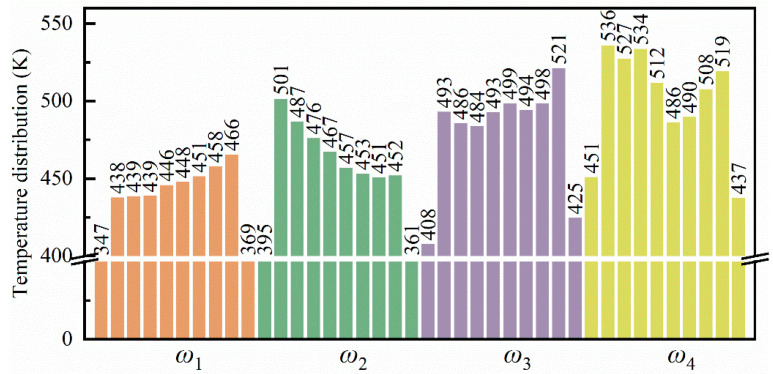
Statistical distribution of average temperature in the leaf spring-type flexible hinge after bending under different bending rates.

Figure 15 illustrates the temperature distribution within the flexure hinge of the leaf spring under varying bending rates. At low bending rates (*ω*_1_ and *ω*_2_), during the 0~2/5 *T* bending phase, no distinct high-temperature zones were observed within the system. However, as the bending degree progressed to the 2/5 *T*~3/5 *T* phase, a small high-temperature area emerged at the bottom of the system, with a temperature range of approximately 350 K to 370 K, as shown in Figure 15(a3). Cao et al. [48] demonstrated that temperature increases are closely associated with material strength degradation, suggesting that elevated temperatures in high-strain zones may heighten the risk of fatigue damage. In these high-temperature regions, atomic motion becomes more vigorous, leading to reduced lattice stability and potentially diminishing the fatigue performance of the material. This thermal effect may further destabilize the microstructure, thereby increasing the risk of fatigue damage. As the bending intensity increased further, high-temperature regions, approximately 470 K to 500 K, formed in areas of significant compressive deformation at the bottom of the system, as shown in Figure 15(a5). With the increasing bending rate, the high-temperature zones expanded gradually within the system, spreading from the specimen sides to areas of pronounced compressive deformation beneath the specimen, with temperatures ranging from 520 K to 590 K, as shown in Figure 15(b5–d5). This pattern indicates that both the extent and distribution of elevated temperatures within the specimen intensified with the increased bending rate and curvature, concentrating in regions of severe compressive deformation. The mean temperature distribution within the system at the extreme bending position under various bending rates is depicted in Figure 16, revealing a pronounced temperature gradient across the system. One to two high-temperature zones were observed, with distinct temperature gradients between the high- and low-temperature regions. As the bending rate increased, both the number of high-temperature zones and the system’s overall average temperature rose from approximately 430 K to 500 K.

## 4. Conclusions

This work, through MD simulation, analyzed the mechanical response, microstructural evolution, and stress distribution of leaf spring-type flexible hinges under various bending rates. The results show that, at lower bending rates (e.g., *ω*_1_ and *ω*_2_), the FCC crystal structure in the system remained relatively stable, with retention rates of 75% and 73%, respectively. The dislocation density remained low (0.8 × 10^15^ m^−2^), the von Mises stress ranged from 4.0 to 4.5 GPa, and the temperature did not exceed 500 K, indicating good structural stability and fatigue resistance, suitable for long-term use. However, at higher bending rates (e.g., *ω*_3_ and *ω*_4_), the FCC crystal structure significantly decreased, with final retention rates dropping to 72% and 71%, accompanied by an increase in HCP and amorphous structures, particularly during the 1/5 *T*~*T* phase, where the HCP structure ratio reached 12%. Meanwhile, dislocation density significantly rose, reaching a peak of 1.8 × 10^15^ m^−2^, the von Mises stress increased to 4.7–5.2 GPa, and the temperature peaked at 590 K, indicating substantially increased plastic deformation and microstructural instability at the higher bending rates, which intensifies stress and temperature concentrations, thereby accelerating fatigue damage. This study demonstrated that the proportion of Shockley and stair-rod dislocations significantly increased at higher bending rates, and the distribution of these dislocations directly influenced the plastic deformation and failure modes of the hinge. Furthermore, variations in the proportions of FCC, HCP, and amorphous structures further affected the macroscopic performance of the flexible hinge. As the bending rates increase, the reduction in FCC structure and the tendency toward amorphization may lead to decreased material stiffness and elasticity, thereby increasing the risk of structural instability and fatigue failure at higher bending rates. The evolution of these microstructures provides critical insights for the optimized design of flexible hinges.

## Figures and Tables

**Table 1:** MD simulation parameters.

Parameters	Value
Material	Single-crystal Cu
Dimensions	100 Å × 500 Å × 250 Å
Number of atoms	1,066,464
Boundary conditions	Periodic boundary condition
Simulation ensemble	NPT, NVE
Potential function	EAM
Bending rate	±2π/2000 rad/ps~±2π/800 rad/ps
Timestep	1 fs

## Data Availability

The data presented in this study are available on request from the corresponding author due to privacy.
